# Ventricular late potential in cardiac syndrome X compared to coronary artery disease

**DOI:** 10.1186/s12872-017-0469-6

**Published:** 2017-01-19

**Authors:** Mohamed Faisal Lutfi

**Affiliations:** Department of Physiology, Faculty of Medicine and Health Sciences, Al-Neelain University, Khartoum, Sudan

**Keywords:** Cardiac syndrome X, Coronary artery disease, Ventricular late potential

## Abstract

**Background:**

Although ventricular late potential (VLP) was extensively studied in risk stratification of myocardial infarction (MI) patients, comparable researches evaluating presence of VLP in MI-free coronary artery disease (CAD) and cardiac syndrome X (CSX) subjects are scarce. This study aimed to compare presence of VLP between CSX and CAD patients.

**Methods:**

Signal average ECG (SAECG) was performed to 49 patients with a history of typical cardiac pain before undergoing diagnostic coronary angiography (DCA) in Al-Shaab cardiac center, Khartoum, Sudan. QRS duration, duration of the terminal part of the QRS complex with amplitude less than 40 microvolts (LAS40) and the root mean square voltage of the terminal 40 milliseconds (RMS40) of the filtered QRS complex were identified for each patient. Presence of two or more of QRS duration > 120 ms, RMS40 > 38 ms and LAS40 < 20 μV was considered indicative of VLP. Associations between VLP and patients grouped according to DCA results were assessed using appropriate statistical tests.

**Results:**

VLP was present in 11.11% (3.63%–24.66%) and 15.38% (2.66%–42.23%) of patients with CAD and CSX respectively. Presence of VLP was comparable in patients with CAD and CSX (OR = 0.69, 95% CI = 0.11–6.05, *P* = 0.692), even after controlling for the possible variations in gender, age, body mass index (BMI), hypertension and diabetes mellitus in the studied groups.

**Conclusion:**

Presence of VLP is comparable among CSX and CAD patients.

## Background

Slow transmission of myocardial depolarization usually forms low amplitude, high frequency waveforms at the end of QRS complex in electrocardiogram (ECG) known as ventricular late potential (VLPs) [1, 2]. Signal average ECG (SAECG) technique is commonly used to improve the resolution of ECG so that the small amplitude VLPs can be measured [3]. VLP is considered as an electrophysiological indicator for reentry ventricular arrhythmia and sudden cardiac death [4]. Although VLP was extensively studied for risk stratification of myocardial infarction (MI) patients [5–7], comparable researches evaluating VLP in MI-free coronary artery disease (CAD) and cardiac syndrome X (CSX) subjects are scarce [8]. CSX is mostly attributed to coronary microvascular ischemia [9] and diagnosed if a subject suffered typical angina attack(s), demonstrated positive cardiac stress test(s) but had normal coronary angiography [10].

Positive VLP was recognized in patients with hypertension [11, 12], diabetes mellitus [13, 14], obesity [15, 16] and other indicators of metabolic syndrome [17, 18]. Bearing in mind that metabolic syndrome is common in patients with CSX [19] as well as CAD [20, 21], it is likely that VLP coexists with these conditions putting the affected patients at risk of ventricular arrhythmia. In theory, this implication is further supported by the fact that myocardial ischemia, whether macrovascular (CAD) or microvascular (CSX), can induce slowing of cardiac depolarization and thus formation of VLP [1–4]. This study aimed to compare presence of VLP between CSX and CAD patients. In addition, influence of gender, age, body mass index (BMI), hypertension and diabetes mellitus on the presence of VLP was assessed among the studied groups.

## Methods

The present study gained ethical approval from ethics review committee (ERC) - Faculty of Medicine - Khartoum University - Sudan. All subjects enrolled in the study signed a written informed consent before being evaluated.

Forty nine patients with a history of typical cardiac pain were evaluated in Al-Shaab cardiac center, Khartoum, Sudan, before undergoing diagnostic coronary angiography (DCA). Past medical history and clinical examination were performed guided by prearranged questionnaire. BMI was calculated by subdividing weight (Kg) by squared height (m^2^). Mean arterial blood pressure (MABP) was estimated from systolic (SBP) and diastolic blood pressure (DBP) by the formula: $\mathrm{MABP} = \mathrm{D}\mathrm{B}\mathrm{P} + \frac{1}{3}\ \left(\mathrm{S}\mathrm{B}\mathrm{P}\hbox{-} \mathrm{D}\mathrm{B}\mathrm{P}\right)$.

DCA demonstrated narrowing of ≥ half of the caliber of ≥ one of the major coronary artery/arteries in 36 of the studied patients and were considered as CAD [22]. The remaining studied patients (*N* = 13) were diagnosed as CSX according to Lee et al criteria [23].

After connecting three orthogonal bipolar electrodes (XYZ lead system), 5-min SAECG was recorded for each patient using Bluetooth ECG transmitter and receiver (DM systems (Beijing) Co. limited - China). The Bluetooth ECG signals received were averaged, amplified and filtered between 25 Hz and 250 Hz. The following SAECG indices were measured: QRS duration, duration of the terminal part of the QRS complex with an amplitude less than 40 microvolts (LAS40) and the root mean square voltage of the terminal 40 milliseconds (RMS40) of the filtered QRS complex. The durations of QRS and LAS40 were measured in milliseconds (ms) while RMS40 was measured in microvolts (μV). QRS duration > 120 ms, RMS40 > 38 ms and LAS40 < 20 μV were considered abnormal [24]. Presence of two or more abnormal SAECG measurements was considered indicative of VLP [24, 25].

Statistical package for the social sciences (SPSS) for windows, version 16.0 (SPSS Inc., Chicago, IL, USA) and OpenEpi calculator (version 2.3) were used for statistical evaluation of the studied parameters. Means, medians, standard deviations (SD), 25 quartile (Q1) and 75 quartile (Q3) were used to describe studied variables based on their distribution curves [26]. Proportions of the studied groups were expressed in percentages (%) and 95% confidence intervals (CI). Means (SD) and median (Q1-Q3) of normally and abnormally distributed variables were compared in the studied groups using Student’s T and Mann-Whitney U tests, respectively. Mid-P exact test was used to evaluate the association between VLP and CAD/CSX. The associations between presence of VLP and CAD/CSX, gender, age, BMI, hypertension and diabetes mellitus were assessed by binary logistic regression analysis. The odds ratio (OR) was used to described the ratio of the odds of an event occurring in patients with CAD to the odds of the same event occurring in subject with CSX. *P* < 0.05 was considered significant.

## Results

CSX patients constituted 26.53% (*N* = 13, 95% CI = 16.21–40.26%) of the subjects who performed DCA (*N* = 49). There remaining patients 73.47% (*N* = 36, 59.74–83.79%) were proved to suffer from CAD. Males constituted 53.85% (29.14–76.79%) and 63.89% (47.58–77.52%) of CSX and CAD patients respectively. Compared with CSX subjects, CAD patients have higher distribution of diabetes mellitus (Chi^2^ = 4.738, *P* = 0.030), significantly increased age (46.69 (6.71) vs. 59.47 (10.31) years, *P* < 0.001), but decreased BMI (29.37 (4.39) vs. 25.83 (4.33) kg/m^2^, *P* = 0.016), Table 1. Distribution of gender (Chi^2^ = 0.406, *P* = 0.524) and hypertension (Chi^2^ = 0,057, *P* = 0.812) were comparable among CSX and CAD patients. N (%) of CAD patient with angiographically defined coronary artery/arteries stenosis are given in Table 2.

**Table 1:** Distribution of age, BMI, MABP and SAECG indices among CSX and CAD patients

	CAD *N* = 36 Mean (SD) Median (Q1 –Q3)	CSX *N* = 13 Mean (SD) Median (Q1 –Q3)	*P*
Age (Years)	58.00 (54.25–67.50)	50.00 (40.00–50.50)	< 0.001
BMI (kg/m2)	25.83 (4.33)	29.37 (4.39)	0.016
MABP (mmHg)	95.43 (14.73)	98.62 (18.15)	0.540
QRS (ms)	84.00 (79.00–104.0)	81.00 (79.00–92.00)	0.403
LAS40 (ms)	31.00 (25.25–34.00)	31.00 (19.00–34.50)	0.777
RMS40 (μV)	113.31 (66.80)	87.64 (51.64)	0.225

**Table 2:** *N* (%) of CAD patients with angiographically defined coronary artery/arteries stenosis/stenoses

Coronary artery	Main branches	*N* (%)
Left coronary artery	Left main coronary trunk	3 (8.33%)
	Anterior descending artery	32 (88.89%)
	Circumflex artery	12 (33.33%)
Right coronary artery	Right main coronary trunk	15 (41.67%)
	Posterior descending artery	2 (5.56%)
	Marginal artery	8 (22.22%)

VLP was present in 11.11% (3.63–24.66%) and 15.38% (2.66–42.23%) of patients with CAD and CSX respectively. Occurrence of VLP was comparable in patients with CAD and CSX (OR = 0.69, 95% CI = 0.11–6.05, *P* = 0.692), Table 3. According to the results of binary logistic regression, the comparable distribution of VLP among CAD and CSX patient persisted after controlling for the possible variations of gender, age, BMI, hypertension and diabetes mellitus in the studied groups, Table 4.

**Table 3:** Association between VLP and CAD/CSX

		VLP Present	VLP Absent	Total
CAD	*N*	4	32	36
	%	11.11%	88.89%	100%
	95% CI	3.63–24.66%	75.34–96.37%	90.36–100%
CSX	*N*	2	11	13
	%	15.38%	84.62%	100%
	95% CI	2.664–42.23%	57.77–97.34%	77.19–100%
Total	*N*	6	43	49
	%	12.24%	87.76%	100%
	95% CI	5.12–23.74%	76.26–94.88%	92.73–100%

Mid-P exact test was used because at least one expected value (row total*column total/grand total) is less than 5, OR = 0.69, 95% CI = 0.11–6.05, *P* = 0.692

**Table 4:** Binary logistic regression analysis of factors associated VLP

	OR (95% CI)	*P*
CAD vs. CSX	0.68 (0.07–6.71)	0.741
Male gender	3.72 (0.36–38.62)	0.272
Age > 50 Years	1.35 (0.13–14.07)	0.803
BMI > 25 Kg/m2	3.88 (0.39–39.14)	0.250
Hypertension	0.61 (0.09–4.17)	0.613
Diabetes Mellitus	0.68 (0.09–5.11)	0.710

## Discussion

It is evident from the results that VLP was present in more than 12% of patients undergoing DCA, being comparably distributed among patients with CAD and CSX. Previous reports demonstrated VLP in 4–6% of healthy subjects [27, 28]. Comparable studies evaluating incidence of VLP in patient with CAD are mostly confined to those with MI [5–7]. To our best of knowledge, the present study is the first to evaluate existence of VLP in patients with CSX. In an old study, positive VLP was documented in 24% of CAD patients with no previous MI and successful thrombolytic therapy received within 4 h of symptom onset [29]. According to Yang et al, the prevalence of VLP in patients with acute MI increased from 32% in the day admission to 52% in the following 7–10 days [6]. Alternatively, Zaman demonstrated positive VLP in 29% of patients with acute MI at the day of admission, 42% after 1 week and 13% after 6 weeks [7]. Compared with the above narrative, the present study confirmed VLP in 11% and 15% of patients with CAD and CSX respectively. Based on our results and previous reports, incidence of VLP among studied patients with CAD and CSX are equally higher than normal subjects [27, 28], but still lower compared with patients with well-established MI [6, 7].

Noteworthy, there is concern about the low positive predictive value of VLP. According to Santangeli et al., the strength of VLP is dependent on its high negative predictive value [3]. However, when positive, VLPs can faithfully stratify the arrhythmic risk of patients in several clinical settings. According to the same study, VLP was highly predictive of cardiac events in patients with ischemic heart diseases, but neither arrhythmogenic right ventricular cardiomyopathy nor syncope of unknown cause. As justified earlier, the aim of our study was to assess risk of VLP in ischemic myocardium whether secondary to macrovascular (CAD) or microvascular (CSX) coronary dysfunction. The comparable distribution of VLP among studied CSX and CAD patients suggests comparable risk of both groups to cardiac events.

Studies on VLP among patients with CAD were mostly evaluating this parameter as a potential predictor of ventricular arrhythmia following myocardial infarction [5, 30]. It was suggested that VLP originate from viable areas around acutely infarcted myocardium [31] or interspersed between fibrous tissues of an old MI [32]. In such conditions, VLP is favored by disturbed architecture of myocardial fibers caused by necrosis and/or fibrosis that ultimately resulted in slow and fragmented depolarization [33]. VLP therefore represent delayed propagation of depolarization waves through a diseased myocardium putting affected patient at higher risk of reentry ventricular arrhythmias [3]. Alternatively, myocardial ischemia induced by microvascular dysfunction is likely to compromise ventricular depolarization and forms the basis for VLP among CSX patients [9]. Old age [17, 18], hypertension [11, 12], diabetes mellitus [13, 14], obesity [15, 16] and other indicators of metabolic syndrome are common in CSX patients [34], and were proved to increase the risk of VLP. Taking into consideration all these risk factors for VLP, it is evident that the tendency of CAD and CSX patients towards abnormal SAECG is comparable. This implication is further supported by our results, which showed not statistical difference in distribution of positive VLP between these groups.

Structural and functional changes that increase the risk of VLP on senescent heart include myocyte hypertrophy, interstitial fibrosis, variation of depolarization propagation velocities and heterogeneity of myocardial refractive periods [35, 36]. Left ventricular hypertrophy and relative myocardial ischemia associated with hypertension may induce slowing of cardiac impulse and predispose to formation of VLP [11, 12]. In experimental animals, Streptozocin induced diabetes mellitus interferes with both cardiac depolarization and repolarization [13]. Autonomic neuropathy associated with insulin resistance gives another explanation for higher risk of diabetic patient to develop VLP [14]. Myocardial injury induced by lipotoxicity [37], parasympathetic withdrawal [38] and insulin resistance [39] are proposed causes of abnormal SAECG in obese subjects [15, 16]. Noteworthy, the comparable distribution of VLP among CAD and CSX patient we studied persisted after we controlled for the possible variations of gender, age, BMI, hypertension and diabetes mellitus in the studied groups using binary logistic regression. Although it is difficult to explain the lack of association between risk factors for VLP and abnormal SAECG findings in the present study, comparable negative findings were demonstrated by another study investigating VLP in patients with ischemic heart disease (IHD) [8]. According to Banasiak et al, comparison of IHD patients with and without VLP showed no discriminating characteristics of the investigated groups, including results of echocardiography, exercise and 24-h Holter ambulatory monitoring [8]. Further investigations are needed to explain why associations between VLP and some of its physiological/pathological determinants are occasionally hidden in patients with coronary macro- or microvascular dysfunctions.

Noteworthy, calculation of appropriate sample size using the formula for unmatched case control study was difficult before collecting the data of this research. For estimation of ideal sample size, the percentages of CAD and CSX patient with positive VLP should be predetermined. However, the percentage CSX patient with positive VLP was not investigated before. Alternatively, different percentages CAD patients with positive VLP were described in the literature but none of them were derived from MI-free CAD patients. The exploratory findings of present study should motivate researchers in the field for more investigations. The sample size of such studies is now easy to determine based on the present findings. Further studies based on properly determined sample size are expected to minimize the disparate number CAD and CSX patients in the present study. Moreover, longitudinal follow-up of patients with CSX and VLP are essential in future studies to prove the prognostic implications of the present findings. Another limitation of the present study is the lack of angina-free healthy control group. Coronary angiography and cardiac stress tests are important for exclusion of CAD and CSX in an ideal control group. Ethically, both tests are difficult to perform in patients with no cardiac complaints, particularly elder individuals. This precluded presence of a control group in the present study.

## Conclusions

The present study is the first to evaluate presence of VLP in patients with CSX. Results showed comparable distribution of VLP among CSX and CAD subjects suggesting a comparable risk of both groups to cardiac events.

## Abbreviations

BMI: Body mass index
CAD: Coronary artery disease
CI: Confidence interval
CSX: Cardiac syndrome X
DBP: Diastolic blood pressure
DCA: Diagnostic coronary angiography
ECG: Electrocardiography
ERC: Ethics review committee
IHD: Ischemic heart disease
LAS40: Duration of the terminal part of the QRS complex with an amplitude less than 40 microvolts
MABP: Mean arterial blood pressure
MI: Myocardial infarction
OR: Odds ratio
Q1: 25 quartile
Q3: 75 quartile
RMS40: Root mean square voltage of the terminal 40 milliseconds of the filtered QRS complex
SAECG: Signal average electrocardiography
SBP: Systolic blood pressure
SD: Standard deviation
SPSS: Statistical package for the social sciences
VLP: Ventricular late potential
